# Different Attentional Patterns for Regret and Disappointment: An Eye‐tracking Study

**DOI:** 10.1002/bdm.1938

**Published:** 2016-02-09

**Authors:** Nadège Bault, Pierre Wydoodt, Giorgio Coricelli

**Affiliations:** ^1^ Center for Mind/Brain Sciences (Cimec) University of Trento Trento Italy; ^2^ Centre for Cognitive Neuroscience CNRS UMR 5229 Lyon France; ^3^ Department of Economics University of Southern California Los Angeles CA USA

**Keywords:** decision making under risk, regret, eye‐tracking, neuroeconomics

## Abstract

The unfavorable comparison between the obtained and expected outcomes of our choices may elicit disappointment. When the comparison is made with the outcome of alternative actions, emotions like regret can serve as a learning signal. Previous work showed that both anticipated disappointment and regret influence decisions. In addition, experienced regret is associated with higher emotional responses than disappointment. Yet it is not clear whether this amplification is due to additive effects of disappointment and regret when the outcomes of alternative actions are available, or whether it reflects the learning feature of regret signals. In this perspective, we used eye‐tracking to measure the visual pattern of information acquisition in a probabilistic lottery task. In the partial feedback condition, only the outcome of the chosen lottery was revealed, while in the complete feedback condition, participants could compare their outcome with that of the non‐chosen lottery, giving them the opportunity to experience regret. During the decision phase, visual patterns of information acquisition were consistent with the assessment of anticipated regret, in addition to a clear assessment of lotteries' expected values. During the feedback phase, subjective ratings and eye‐tracking results confirmed that participants compared their outcome with the outcome of the non‐chosen lottery in the complete feedback condition, particularly after a loss, and ignored the non‐realized outcome of the chosen option. Moreover, participants who made more visual saccades consistent with counterfactual comparisons during the feedback period anticipated regret more in their decisions. These results are consistent with the proposed adaptive function of regret. © 2016 The Authors Journal of Behavioral Decision Making Published by John Wiley & Sons Ltd.

## Introduction

When we choose among alternatives, we may have the opportunity to compare the consequences of our choices with the consequences of non‐chosen options. The unfavorable comparison between obtained and foregone outcomes can generate error signals, called regret following the economics (Bell, 1982; Loomes & Sugden, 1982) and psychology tradition (Zeelenberg, van Dijk, Manstead, & van der Pligt, 2000), or fictive errors (Lohrenz, McCabe, Camerer, & Montague, 2007) in neuroscience literature. Regret and disappointment are experienced in response to an unfavorable outcome of a decision, and both are related to a process of counterfactual thinking in which the obtained outcome is compared with an outcome that might have been (Zeelenberg, van Dijk, & Manstead, 1998). Counterfactual thinking influences individuals' emotional reactions—in terms of valence and intensity—to outcomes and events (Boles & Messick, 1995; Kahneman & Miller, 1986). Several aspects may differentiate regret and disappointment. According to Zeelenberg et al. (1998), whereas regret is related to behavior‐focused counterfactual thought in which the decision maker's own actions are the elements of the counterfactual, disappointment is related to situation‐focused counterfactual thought in which aspects of the situation are changed. Contrary to disappointment, regret is associated with a feeling of responsibility and may have a more important role in learning to evaluate our actions (Camille et al., 2004).

Mellers, Schwartz, and Ritov (1999) designed the “regret gambling task,” to study the effect of feedback information about the outcome of foregone alternatives on the evaluation of the obtained outcome. In the partial feedback condition, where only the feedback of the chosen lottery was provided, emotional ratings increased with the obtained outcome and decreased as a function of the chosen lottery's unobtained outcome (i.e., intra‐lottery comparison), thus characterizing the disappointment effect. A win (e.g., of $8) was perceived as more pleasurable when compared with a missed loss (e.g., of $32) rather than a missed larger gain (e.g., of $32). In the complete feedback condition, the obtained outcome was compared with that of the unselected lottery (i.e., inter‐lottery comparison), and this counterfactual comparison modulated self‐reported emotional responses, interpreted by the author as reflecting a regret effect. Camille et al. (2004) replicated these findings using the same task and showed that the influence of regret on outcome evaluation was amplified compared with disappointment. Moreover, regret was associated with stronger emotional arousal, as indexed by skin conductance responses, than disappointment. In addition to regret and disappointment effects, Camille et al. (2004) reported an effect of the non‐realized outcome of the chosen gamble in the complete feedback condition (disappointment in regret). Therefore, in the complete feedback condition, two reference points can be taken into account in the evaluation of an outcome: the outcome of the non‐chosen lottery and the non‐realized outcome of the chosen lottery. If both types of counterfactual outcomes do influence the subjective evaluation of one's decision outcomes, then amplified feeling of regret in the complete condition might simply be interpreted as an additive effect of regret and disappointment. This may run counter to the interpretation of the amplified feeling of regret as a distinct learning signal; interpretation that holds only if the first type of counterfactual outcome is taken as a reference point. It is not straightforward, however, to disentangle the differential effects of both forms of missed outcomes on the subjective evaluation of the obtained outcome. From this perspective, investigating the pattern of visual inspection of the lotteries during the feedback period may prove useful to infer the underlying processes of outcome evaluation (Johnson, Schulte‐Mecklenbeck, & Willemsen, 2008).

We used a similar lottery task with partial and complete feedback in combination with eye‐tracking to investigate, on the one hand, the link between fixations and choice behavior during the decision phase and, on the other hand, fixations on obtained and forgone outcomes during the feedback phase. In the vein of recent research in the field of decision neuroscience, we based our work on the assumption that visual attention plays an active role in shaping emotional experiences and in constructing decisions (Orquin & Mueller Loose, 2013). We describe decision processes considering the nature and the pattern of visual information acquired by participants, as measured by eye‐tracking. To our knowledge, this is the first study investigating patterns of information acquisition related to the anticipation of regret in decision under risk.

Two different visual attention patterns are possible depending on whether the emotional amplification of regret is due to a feeling of responsibility or to an additive effect of disappointment and regret. In the first case, we hypothesize that when the outcome of the non‐chosen lottery is revealed, in the complete feedback condition, this monetary amount will be attended longer than in the partial feedback condition. Concomitantly, the attention toward the non‐realized outcome of the chosen lottery will be reduced. By contrast, the additive hypothesis would require no change in visual attention toward the non‐realized outcome of the chosen lottery in the complete feedback condition compared with the partial feedback condition, and possibly no difference between the two forms of counterfactual information.

When considering decision mechanisms, Camille et al. (2004) proposed a model of choice that incorporates anticipated regret and disappointment in addition to maximizing expected earnings. This approach has been successful in explaining choices in several other datasets (Coricelli et al., 2005; Larquet, Coricelli, Opolczynski, & Thibaut, 2010; Simioni et al., 2012). Anticipating regret versus evaluating expected values (in order to maximize earnings) in choices implies several differences in terms of visual patterns of information acquisition. To evaluate the expected values of a lottery pair, one will integrate information related to the monetary payoffs and respective probabilities of a single lottery and then switch to the other lottery. This was for instance the pattern of looks‐up related to choices in Glöckner and Herbold (2011) and in the easy condition of Arieli, Ben‐Ami, and Rubinstein (2011). In that study, when the computation of the expected value was easy, participants attended the prospective payoffs and associated probabilities of each lottery separately. By contrast, the regret theory states that anticipating regret relies on the computation of a relative utility function. The utility associated with an option from a choice set depends not only on the expected consequences of that option,but also on those of the alternative options (refer to section on “Theoretical framework: behavioral and attentional pattern of regret‐based decision making”). Thus, we hypothesize that anticipating potential regret will require one to make more comparisons between lotteries and especially to compare the lowest monetary amount of each lottery with the highest amount of the other. The aim of the study was also to provide important insights into the role of experienced emotions in modulating attention in subsequent choices, thus providing the first evidence of the role of regret‐driven attention in decision making. We hypothesize that the more participants attend the outcome of the non‐chosen lottery, the more they will anticipate regret in subsequent choices.

### Theoretical framework: behavioral and attentional pattern of regret‐based decision making

#### Experienced utility (outcome phase of the choice process)

We consider a decision maker who observes both the outcome obtained from his or her choice and the counterfactual outcome, an outcome he or she could have had alternatively. The difference between these two terms defines a relative experienced utility. The relative experienced utility captures the importance of the comparison of the obtained outcome versus the counterfactual outcome. How important relative experienced utility is for the well‐being of the decision maker potentially depends on the nature of the counterfactual outcome. Our experimental paradigm allows us to distinguish between the role of chance and personal responsibility. In the partial feedback condition, a counterfactual outcome is the outcome that the decision maker would have had by a different resolution of uncertainty. Disappointment is elicited by the unfavorable comparison between the obtained and non‐realized outcomes of the chosen lottery. In the complete feedback condition, however, a counterfactual outcome is what he or she could have had with a different choice, in addition to the obtained outcome. Results from previous studies (Camille et al., 2004) showed that (i) counterfactual comparison influence outcome evaluation in both partial and complete feedback conditions and (ii) the regret effect in complete feedback condition is more intense than disappointment in partial feedback condition (i.e., amplification effect).

#### Decision utility (decision phase of the choice process)

To evaluate lotteries ex ante, before choice is made, a decision maker will anticipate the future disappointment associated with a bad resolution of the chosen option and the future regret associated with a bad decision. As disappointment is elicited by the unfavorable comparison between a decision outcome and non‐realized outcomes of the chosen option, anticipated disappointment may be related to the distance between the possible outcomes of the considered option. Regret is elicited by the unfavorable comparison between a decision outcome and the outcome of a rejected alternative option. Whereas future disappointment is evaluated by considering the elements of the attended lottery only, anticipating future regret involves comparing the possible outcomes of all available options. As a consequence, anticipating disappointment and regret in choice will require different attentional patterns of information integration. Specifically, taking into account future regret in decision will translate in making more comparisons between the possible outcomes of different options, thus more inter‐lottery saccades.

#### Effect of experienced regret on decision utility

Experienced and decision utilities are strongly linked. The main hypothesis is that the experience of regret will result in more anticipation of this emotion during subsequent choices. To verify this hypothesis, we tested the effect of experienced regret in early trials on choice behavior (i.e., anticipation of regret) in late trials of our experiment.

#### Behavior and visual attention

We state the following hypotheses for correspondence between behavioral and eye‐tracking effects in the context of a choice between two lotteries: (i) after the outcomes of both lotteries are revealed, greater attention is paid to the outcome of the unchosen lottery than to the non‐realized outcome of the chosen lottery (i.e., the amplification effect is associated with more attention toward the counterfactual outcome of the unchosen lottery). This concerns the personal responsibility effect, which states that the counterfactual comparison is more important in the case of personal responsibility rather than chance. Intuitively, the subject is more affected in terms of relative utility if the counterfactual comparison is due to his or her choice rather than simply pure chance. (ii) During the decision phase, regret‐based choices induce more inter‐lottery comparisons (transitions of visual attention from one lottery to another). (iii) The experience of regret after feedback induces anticipation of regret in subsequent choices, as indexed by a pattern of visual attention associated with more inter‐lottery comparisons.

## Methods

### Participants

Twenty healthy adults (11 men and nine men, *M*_age_ = 22.5 years, age range: 19–25 years) with normal vision participated in the study approved by the French National Ethical Committee. Participants were recruited through advertising posters in universities in Lyon.

### Task

A lottery task was used, manipulating the magnitude and probabilities of potential gains and losses. Subjects repeatedly chose between two lotteries, with the aim of maximizing their earnings. Each lottery had two possible outcomes from the set of values {−20; −5; +5; +20} with probabilities of the first outcome taken from the set {.2; .5; .8}. We ensured that the difference in expected values of the two lotteries of all pairs did not exceed 7 euros, so none of the two lotteries clearly dominated the other (Table S1).

The highest possible outcome of each lottery was always positioned on the left side and the lowest possible outcome on the right side. To control for stimuli saliency, the colors used to depict the potential outcomes and their respective probabilities (orange and blue) had comparable saturation (169 and 173, respectively) and luminance (130 and 136). To ensure discrimination of visual fixations on the diverse elements of the lotteries, the digits representing the possible wins and losses were separated from each other, and from the sector on the circle representing the associated probability, with an angle of 9°.

### Experimental procedures

Participants underwent 80 trials in two successive sessions. Two sequences of trials were undertaken by each participant: one sequence with partial feedback (40 trials) and one with complete feedback (40 trials). Eleven participants experienced the partial feedback condition first, and nine participants started with the complete feedback condition. In the partial feedback condition, only the outcome of the chosen lottery was revealed to the participant; in the complete feedback condition, the outcome of both the chosen and non‐chosen lotteries were revealed at the same time. Participants thus had the opportunity to compare their outcome to what they could have obtained, had they chosen the alternative lottery.

**Figure 1:**
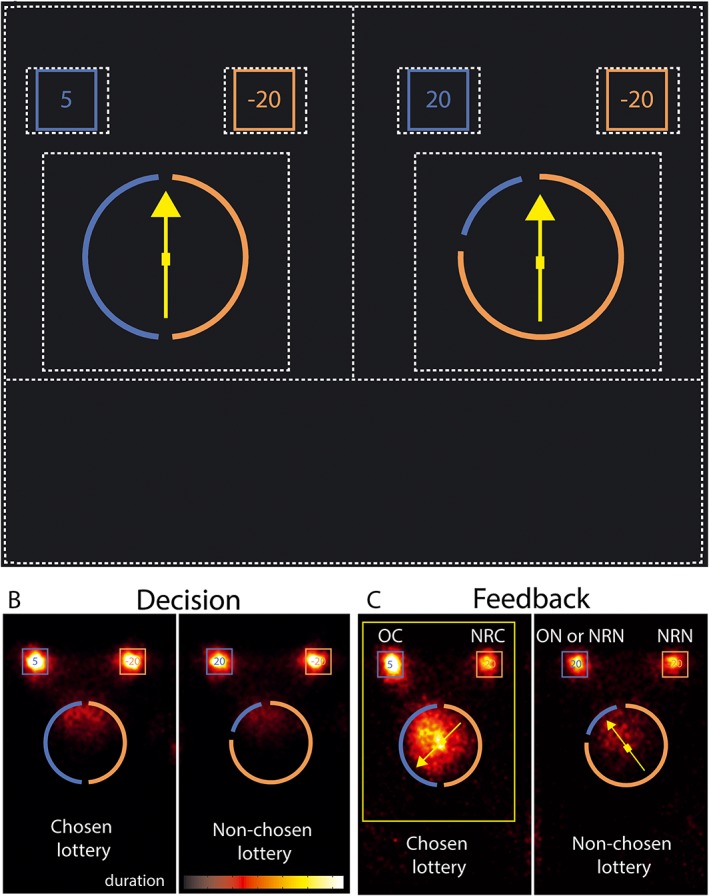
(A) Example of two lotteries presented at the beginning of the trial. The portions of each arc represent the probability associated with the payoffs depicted in the square box above each portion. Here, the left lottery has a 50% chance of earning 5 euros and a 50% chance of losing 20 euros. The right lottery has a 20% chance of earning 20 euros and an 80% chance of losing 20 euros. Probabilities can take values {.5, .2, .8}, and payoffs can take any value within {−20, −5, +5, +20}.The dotted lines represent the AOIs considered for the analyses, one large AOI for each lottery one for each monetary amount and for each probability wheel. (B) Distribution of fixation points on the chosen and non‐chosen lotteries during the decision period, weighted by the fixation duration, for all trials and participants. The fixation points are plotted on one example pair of gambles: 58.74% of the total number of fixations occurred in the monetary amounts AOIs and 23.20% in the wheels AOIs. (C) Distribution of fixation points on chosen and non‐chosen lotteries, obtained and non‐realized outcomes during the feedback period, weighted by the fixation duration, for all trials and participants. OC, obtained‐chosen; NRC, non‐realized‐chosen; ON, outcome‐non‐chosen; NRN, non‐realized‐non‐chosen. Of the fixations, 34.27% occurred in the monetary amounts AOIs and 48.10% in the wheels AOIs; 5.60% of the fixations were located in the place of the future location of the emotional scale, in anticipation of its appearance

At the beginning of the trial, two lotteries were displayed (Figure 1). The subject could choose one of the two lotteries at any time by pressing one of two arrow keys on a keyboard (self‐paced). After the participant had made their choice, they saw their choice for 2 seconds, then an arrow spinning for 5 seconds, and the outcome for 3 seconds. At the end of the trial, the participant was asked to provide a subjective emotional rating on the outcome of their choice (i.e., “How do you feel about the outcome of your choice”) on a scale from −50 (*extremely negative*) through 0 (*neither positive nor negative*) up to +50 (*extremely positive*). Trials were separated by a 5‐second black screen.

#### Payments

Participants were financially motivated. So as to avoid participants mentally summing their earnings and to incentivize them to treat trials independently, participants were told that the results of 10 randomly selected trials would be summed at the end of the experiment and that they would receive this amount in addition to a 5‐euro show‐up fee. The French National Ethical Committee required that all participants received the same monetary compensation; therefore, they were paid 15 euros at the end of the experiment. At the end of the experimental session, participants were debriefed regarding the goals of the study and the use of deceptive monetary incentive. In order to avoid any transmission of knowledge about the deceptive payment procedure between participants, we recruited them in various universities building across Lyon, and we did not include individuals who learned about the study through former participants.

### Data analyses

#### Choice behavior analyses

Choice behavior was analyzed based on panel data analysis, using the statistical software package stata (StataCorp, College Station, TX, USA). We ran mixed (panel data) logit regressions, which took each participant as the unit and the trial as time, and estimated both random and conditional fixed effects. Parameters were estimated by maximum likelihood.

Given that *Pr*(*g*_1_) = 1 − *Pr*(*g*_2_), where *Pr*(*g*_1_) and *Pr*(*g*_2_) are the probabilities of choosing lottery 1 and lottery 2, respectively, we defined the probability of choosing *g*_1_ in terms of three factors affecting the choice: expected value (*dEV*), anticipated regret (*r*), and anticipated disappointment (*d*). *x*_1_, *y*_1_ and *x*_2_, *y*_2_ are the two possible outcomes of the first (*g*_1_) and second (*g*_2_) lotteries, respectively, with *x*_1_ *> y*_1_, and *x*_2_ *> y*_2_. The probability of *x*_1_ is *p*, and the probability of *y*_1_ is (1 − *p*). The probability of *x*_2_ is *q*, and the probability of *y*_2_ is (1 − *q*).

The model is *Pr*(*g*_1*t*_) = *F* [*dEV_it_*, *r_t_*, *d_t_*], where *t* is time. We ran a mixed‐effect logistic regression to estimate the probability of the participant choosing lottery 1, as a function of the difference in expected value (*dEV*), anticipated regret (*r*), and disappointment (*d*), such as

$$Pr\left(c=1\left|dEV,r,d\right.\right)=\frac{1}{1+e^{\left(\alpha +\beta \cdot dEV\;+\;\gamma \cdot r\;+\;\delta \cdot d\right)}}$$

The dependent variable, “choice of *g*_1_,” is 1 when the subject chooses *g*_1_ and 0 when the subject chooses *g*_2_. Independent variables are *d*, *r*, and *dEV*. The difference in expected value when choosing *g*_1_ is defined as

$$\begin{array}{c} dEV=EV_{1}-EV_{2}\\ =\left[p\cdot x_{1}+\left(1-p\right)y_{1}\right]-\left[q\cdot x_{2}+\left(1-q\right)y_{2}\right] \end{array}$$

A positive (negative) and significant expected value (*dEV*) coefficient indicates that subjects consistently choose the lottery with the highest (lowest) expected value.

Anticipated disappointment when choosing *g*_1_ is defined as

$$\begin{array}{c} d=d_{2}-d_{1}\\ =\left[\left|y_{2}-x_{2}\right|\left(1-q\right)\right]-\left[\left|y_{1}-x_{1}\right|\left(1-p\right)\right] \end{array}$$

Anticipated regret when choosing *g*_1_ is defined as

$$r=\left|y_{2}-x_{1}\right|-\left|y_{1}-x_{2}\right|$$

Anticipated regret is based on the consideration of a possible choice alongside the rejection of its alternatives. The process of minimizing the anticipated regret (denoted as *r*) consists of rejecting the lottery associated with the highest regret propensity, when comparing the lowest outcome of this lottery and the highest outcome of the alternative lottery.

Positive coefficients for *r* and *d* indicate that subjects consistently anticipated (minimized) regret and disappointment, respectively. To detect multicollinearity, we computed the variance inflation factor of the model. The mean variance inflation factor is 2.04 (range 1.68–2.63), suggesting very low variance inflation.

Anticipated disappointment depends on the spread out of the lotteries' possible outcomes, which is similar to a measure of risk propensity. We also report a regression in which anticipated disappointment is replaced by a more conventional measure of risk. Risk propensity when choosing *g*_1_ is defined as

$$\begin{array}{c} dsd=sd_{1}-sd_{2}\\ =\sqrt{p{\left(x_{1}-EV_{1}\right)}^{2}+\left(1-p\right){\left(y_{1}-EV_{1}\right)}^{2}}-\sqrt{q{\left(x_{2}-EV_{2}\right)}^{2}+\left(1-q\right){\left(y_{2}-EV_{2}\right)}^{2}} \end{array}$$

The mean variance inflation factor of this model is 1.21 (range 1.13–1.26).

We also tested the hypothesis that participants anticipate regret more for pairs of lotteries with a smaller difference in expected values. We estimated with a mixed‐effect logistic regression the probability of choosing the lottery, minimizing regret as a function of |*EV*_1_ − *EV*_2_|.

Following this analysis, we categorized trials into regret‐driven and *dEV*‐driven decisions. We first isolated lottery pairs for which *dEV* and *r* had opposite signs, meaning that maximizing expected values or minimizing regret results in opposite choices. Among these pairs of lotteries, a trial was considered as regret driven when participants chose the lottery associated with the lowest potential regret and with the lowest expected value. The rest of the trials—when participants maximized expected values regardless of regret (including pairs of lotteries for which *dEV* and *r* had the same sign)—were categorized as *dEV*‐driven trials.

#### Affective report analyses

We compared how combinations of obtained and foregone outcomes modulated mean affective ratings in the two conditions, partial and complete feedback, during the feedback phase. Events were classified according to their feedback condition and to whether or not the participant experienced a relative loss or a relative gain. Trials were categorized as relative gain trials if the counterfactual comparison was advantageous and as loss trials if it was disadvantageous, regardless of the sign of the obtained outcome. In the partial feedback condition, the participant could experience disappointment in case of relative loss, that is, when the obtained outcome is worse than the non‐realized outcome of the selected lottery, or joy in case of relative gain, when the obtained outcome is better than the non‐realized outcome of the selected lottery (i.e., intra‐lottery comparison). In the complete feedback condition, where information about the outcome of the non‐chosen lottery is available, the participant can experience regret when the obtained outcome is worse than the outcome of the unselected lottery or relief when the obtained outcome is better than the outcome of the unselected lottery (i.e., inter‐lotteries comparison). In addition, with complete feedback, comparison between the obtained outcome and the non‐realized outcome of the chosen lottery is still possible (intra‐lottery comparison). The latter event will be considered in the analyses.

We used the following labels in our analyses: the obtained outcome of the chosen lottery is labeled OC and the non‐realized outcome of the chosen lottery is NRC. In the partial feedback condition, NRN1 and NRN2 are the two possible outcomes of the non‐chosen lottery (with NRN1 > NRN2). In the complete feedback condition, ON is the outcome of the non‐chosen lottery and NRN the non‐realized outcome of the non‐chosen lottery.

The statistical analyses were conducted with the statistical software package stata (StataCorp; Release 9/SE). Nonparametric tests were applied to the datasets because the data violated several parametric assumptions, particularly distribution normality as the affective scale is truncated. The significance of the difference between behavioral variables, such as subjective evaluations, is estimated with the Wilcoxon signed‐rank test (WSRT, nonparametric test); the hypothesis tested is that the distribution of two random variables for matched pairs is the same. A mixed linear regression was run in the complete feedback condition (800 observations) to confirm results from comparison of mean affective ratings. This regression took the affective rating in each trial as the dependent variable and the obtained and non‐realized outcomes of the chosen lottery as well as the outcome and non‐realized outcome of the non‐chosen lottery as predictors.

#### Eye‐tracking recordings and analyses

We used the video‐based Tobii 1750 eye‐tracker with a 1280 × 1024 pixel screen. The table‐mounted camera and IR light system perform a 50‐Hz sampling rate acquisition and computes position for both eyes. Participants were sitting approximately 50 cm from the screen, and their head was free of movement. A pre‐analysis using clearview software (ClearView 2.6.0 analysis software 2007, Tobii AB, Danderyd, Sweden) divided eye movements into fixations and saccades. Fixation points were defined as gaze pause when remaining within a 30 × 30 pixel square for at least 40 milliseconds. Fixation points accounted for 67.9% of all data points. Additional analysis steps were performed with matlab MathWorks, Natick, MA, USA. Parametric tests were applied because the data did not violate parametric assumptions. We focused on the decision (self‐paced) and feedback (3 seconds) periods. The trajectories and fixation points were grouped in pre‐defined areas of interest (AOIs) corresponding to stimulus regions. We defined one AOI for each entire lottery, plus one for each of the four monetary amounts, and one for the wheel representing the probabilities of obtaining the potential outcomes (Figure 1B and C). For decision and feedback events, we considered the number of fixations on each AOI, that is, the number of times the gaze entered the AOI and the total fixation time for each AOI. A fixation may include several fixation points within the same AOI; however, fixation times and the fixation point count are highly redundant measures. As fixation times might be more informative of the information extraction process, the number of fixation points will be not considered in the study. For the decision periods, in addition to comparing the total fixation times, we investigated the information acquisition pattern by computing a transition matrix. The purpose of this matrix is to describe the main patterns of transition between the AOIs by computing the relative frequencies of these transitions. For each AOI *i*, the number of transitions—defined as two successive fixations—from that AOI*_i_* to each of the other AOI was computed. The relative frequency of transition was computed by dividing the number of transition from AOI*_i_* to AOI*_j_* with the total number of transitions from AOI*_i_* to any other AOI.

## Results

### Feedback phase

#### Subjective outcome evaluation

**Figure 2:**
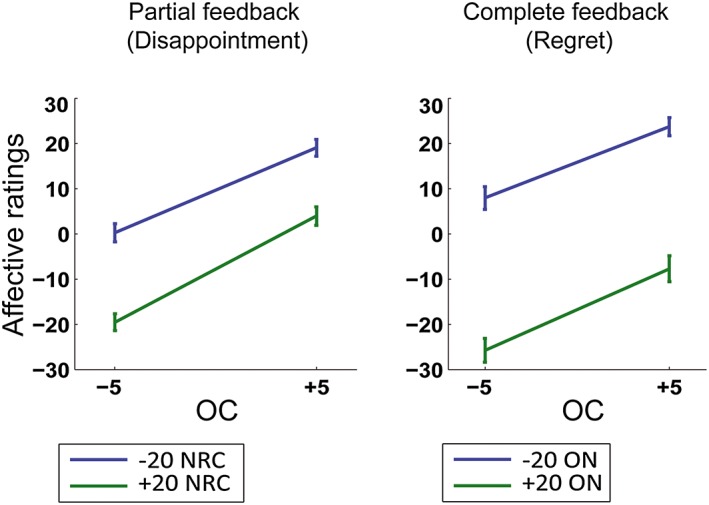
Affective ratings in the partial and complete conditions. In the partial condition, the mean affective ratings of the obtained outcome of the chosen lottery (OC) are depicted as a function of the non‐realized outcome of the chosen lottery (NRC). In the complete condition, it is depicted as a function of the outcome of the non‐chosen lottery. Error bars stand for standard error of the mean

Participants evaluated their outcome more negatively in the regret events (i.e., OC < ON in the complete feedback condition, average rating = −21.40, *SEM =* 2.51) than in the disappointment events (OC < NRC in the partial feedback condition, average rating = −17.35, *SEM =* 1.96; WSRT, *Z* = 1.941, *p* < .05, Cohen's *d* = .40). No difference was found between the relief (OC > ON in the complete feedback condition, average rating = 24.78, *SEM =* 2.15) and rejoice (OC > NRC in the partial feedback condition, average rating = −24.59, *SEM =* 2.07) events. Following Camille et al. (2004), we looked at specific pairs of obtained/unobtained outcomes to confirm that the regret effect was not driven by a difference in mean gains and losses between the partial and complete feedback conditions (Figure 2). In the partial feedback condition, a loss of 5 euros was perceived as more unpleasant and a gain of the same value as less pleasant when the non‐realized outcome from the same lottery (NRC) was a gain of 20 euros instead of a loss of 20 euros (WSRT, *Z* = 3.921, *p* < .001, *d* = 1.50, for −5 obtained; *Z* = 3.702, *p* < .001, *d* = 1.31, for +5 obtained). In the complete feedback condition, emotional reactions were strongly modulated by the outcome of the unchosen alternative (ON), showing the effect of regret (WSRT, *Z* = 3.823, *p* < .001, *d* = 1.65, for −5 obtained; *Z* = 3.823, *p* < .001, *d* = 1.64, for +5 obtained). Direct comparisons between the two feedback conditions revealed that an unobtained outcome of 20 euros resulted in more negative emotional ratings in the complete feedback condition (WSRT, *Z* = 2.535, *p* < .05, *d* = .58, for −5 obtained; *Z* = 2.375, *p* < .001, *d* = .90, for +5 obtained), reflecting more intense feelings of regret than disappointment. A linear regression in the complete feedback condition (number of observations = 800, Wald *χ* ^2^ = 3067.65, *p* < .0001) revealed that subjective ratings improved with the obtained outcome (OC, *β* = 1.39, *p* < .001) and worsened with both the non‐realized outcome of the chosen lottery (NRC, *β* = −.18, *p* < .001) and the outcome of the non‐chosen lottery (ON, *β* = −.52, *p* < .001). The effect of the non‐realized outcome of the non‐chosen lottery (NRN) was not significant (*β* = −.04, *p* = .337). Note that the effect of ON on subjective ratings is almost three times higher than that of NRC. Given that NRC and ON show a relatively high correlation, it is difficult to draw any conclusion on the effect of NRC in the complete feedback condition or, put differently, on the role of disappointment in regret situations, based on the analysis of subjective ratings alone. We thus turned to eye‐tracking data to investigate whether participants will still attend the NRC outcome in the complete feedback condition.

#### Eye‐tracking

After the outcome of the lotteries had been revealed, participants fixated on the chosen lottery longer (*M* = 1365.25 milliseconds, *SEM =* 71.64) and more often (*M* = 1.86, *SEM =* .04) than the non‐chosen one (total fixation time: *M* = 628.17 milliseconds, *SEM =* 44.83; number of gaze entries into AOI: *M* = 1.18, *SEM =* .05). A two‐way analysis of variance revealed a main effect for choice (chosen versus non‐chosen lottery, total fixation time: *F*(1, 19) = 147.21, *p* < .001, *d* = 1.68; number of gaze entries into AOI: *F*(1, 19) = 398.59, *p* < .001, *d* = 1.73), a main effect for the condition (only significant for the number of gaze entries into AOI: *F*(1, 19) = 57.19, *p* < .001, *d* = 1.34) and a significant interaction between choice and feedback condition (total fixation time: *F*(1, 19) = 56.65, *p* < .001; number of gaze entries into AOI: *F*(1, 19) = 36.81, *p* < .001). In the complete feedback condition (*M* = 854.12 milliseconds, *SEM =* 42.55), participants looked at the non‐chosen lottery twice as long as in the partial feedback condition (*M* = 402.22 milliseconds, *SEM =* 47.11), and this difference was highly significant (*t*(19) = 8.84, *p* < .001, *d* = 1.49).

**Figure 3:**
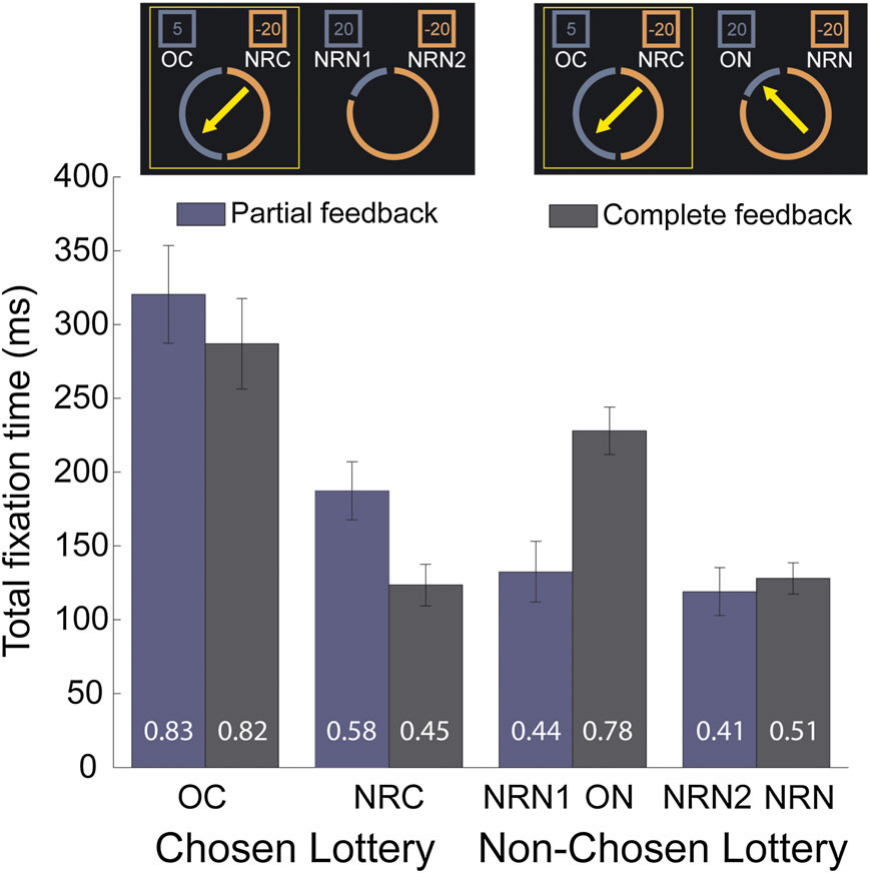
Total duration of fixations on the four outcomes in partial and complete conditions during the feedback phase. The four monetary amounts are categorized depending on their relevance for the participant after the outcome of the chosen (in the partial feedback condition), or both lotteries (complete feedback condition) have been revealed. The values shown on the bars represent the proportion of trials with at least one fixation in the AOI. OC, obtained‐chosen; NRC, non‐realized‐chosen; ON, outcome‐non‐chosen; NRN, non‐realized‐non‐chosen. In the partial feedback condition, NRN1 > NRN2. OC, NRC, and ON can take any value within {−20, −5, +5, +20}, depending on the participant's choice and on the result of the lotteries. Error bars stand for standard error of the mean

Results from emotional ratings suggest that regret is elicited by the comparison between the obtained outcome and the outcome of the non‐chosen lottery. Eye‐tracking data confirmed the importance of the outcome of the non‐chosen lottery for outcome evaluation (Figure 3). Under both feedback conditions, participants primarily looked at their obtained outcome (OC) the longest. In the partial feedback condition, they looked at the non‐realized outcome of the chosen lottery (NRC: *M* = .78, *SEM =* .07) more often than at the two possible outcomes of the other lottery (NRN1: *M* = .55, *SEM =* .06; *t*(19) = 3.28, *p* < .005, *d* = .70; and NRN2: *M* = .49, *SEM =* .05; *t*(19) = 4.67, *p* < .001, *d* = .93). The difference in total fixation time between NRC and both NRN1 and NRN2 was significant (*t*(19) = 2.54, *p* < .05, *d* = .59; and *t*(19) = 3.77, respectively, *p* < .001, *d* = .78), but there was no difference between fixation times for the two non‐realized outcomes of the non‐chosen lottery (*p* = .198). The distribution of fixation times on the monetary amounts was very different in the complete feedback condition. Participants attended their obtained outcome most (OC, number of gaze entry into AOI: *M* = 1.12, *SEM =* .07), with the second most attended outcome being that of the non‐chosen lottery (ON, number of gaze entry into AOI: *M* = .95, *SEM =* .06). They looked more often at ON than NRC (*M* = .57, *SEM =* .05; *t*(19) = 4.69, *p* < .001, *d* = 1.22) and NRN (*M* = .61, *SEM =* .05; *t*(19) = 7.03, *p* < .001, *d* = 1.15), and they spent significantly more time looking at ON than at NRC (*t*(19) = 4.90, *p* < .001, *d* = 1.22) and NRN (*t*(19) = 8.96, *p* < .001, *d* = 1.27). NRC was fixated in only 45% of the trials, whereas ON was fixated in the 78% of the trials (*t*(19) = 6.26, *p* < .001). This is contrary to the partial feedback condition, in which the number of discrete fixations (*p* = .313) and fixation time (*p* = .746) on NRC was not higher than the fixation time on NRN, suggesting that participants stopped attending the non‐realized outcome of the lottery they had chosen when the feedback from the other lottery was available. This finding runs counter to the argument that the unobtained outcome of the chosen lottery (NRC) has a real impact on subjective ratings in complete feedback condition, showing a different pattern of visual attention for regret compared with disappointment.

### Decision phase

#### Choice behavior

Participants took more time to make their choice in the complete feedback condition (*M* = 5.72 seconds, *SEM =* 1.79) than in the partial feedback condition (*M* = 5.25 seconds, *SEM =* 1.51; *t*(19) = 2.02, *p* < .05, *d* = .28). They chose according to the expected values of the lotteries (Table 1, the coefficient for *dEV* is positive and significant). In addition, anticipated regret and disappointment were predictive of choices. Importantly, the effect of anticipated disappointment on decisions was significant in the partial feedback condition, but it was not in the complete feedback condition. Note, however, that directly testing the interaction between the feedback condition and regret or disappointment did not reveal a significant effect. In the complete feedback condition, the significant negative interaction between *dEV* and *r* suggests that the probability of minimizing regret increased when the difference in expected values between lotteries decreased. This effect was confirmed by a logit mixed‐effect regression (Wald *χ* ^2^ = 95.09, *p* < .001, *β* = −.228, *p* < .001). In other words, participants relied more on anticipated regret as a decision variable when the lotteries were harder to discriminate on the basis of their expected values. The results were very similar when considering risk propensity instead of anticipated disappointment (Table S2).

**Table 1:** Mixed logistic regression analysis modeling choices in the partial and complete feedback conditions

Variables	Partial feedback				Complete feedback			
	Coeff	*SE*	*z*	*p*	Coeff	*SE*	*z*	*p*
Diff in expected values (*dEV*)	.1519	.0303	5.02	<.001	.1886	.0312	6.04	<.001
Anticipated disappointment (*d*)	.0225	.0098	2.29	.022	.0148	.0100	1.47	.140
Anticipated regret (*r*)	.0278	.0067	4.15	<.001	.0309	.0068	4.53	<.001
Interaction *dEV* × *r*	−.0033	.0023	−1.41	.159	−.0053	.0024	−2.17	.030
Interaction *dEV* × *d*	−.0045	.0026	−1.7	.088	−.0022	.0026	−.83	.408
Interaction *d* × *r*	.0007	.0004	1.72	.086	.0003	.0004	.64	.521
Interaction *dEV* × *r* × *d*	−.0001	.0002	−.4	.690	.0000	.0002	−.05	.961
Constant	.1785	.1109	1.61	.107	−.0025	.1092	−.02	.981
	Log likelihood = −501.75923				Log likelihood = −494.20136			
	Wald *χ* ^2^(7) = 91.35				Wald *χ* ^2^(7) = 101.98			
	Prob > *χ* ^2^ = .0000				Prob > *χ* ^2^ = .0000			

The probability of choosing the left lottery over the right one is estimated as a function of the difference in expected values between the two lotteries, anticipated regret and disappointment. The regression includes the interactions of these three variables with one another.

#### Eye‐tracking

During the decision phase, participants mainly looked at the four monetary amounts and at the intersection between the different portions of the wheel representing the probabilities (Figure 1B). They looked at each monetary amount 2.58 times (*SEM =* .18) for 655.94 milliseconds (*SEM =* 58.28) on average per trial. Each probability wheel was fixated on 1.90 times (*SEM =* .14) for 429.06 milliseconds (*SEM =* 42.68). They looked more often and longer at the lottery they were about to choose (number of gaze entries into AOI: *M* = 3.02, *SEM =* .19; total fixation time inside the AOI: *M* = 2.34 seconds, *SEM =* .18) than at the subsequently rejected lottery (number of gaze entries into AOI: *M* = 2.71, *SEM =* .18; duration: *M* = 1.83 seconds, *SEM =* .14), and this difference was significant (number of gaze entries into AOI: *t*(19) = 12.80, *p* < .001, *d* = 1.05, time spent inside the AOI: *F*(1, 19) = 123.92, *p* < .001, *d* = .43).

Visual inspection strategies of available information may reveal how *dEV* and *r* are computed as decision criterions. Particularly, considering the expected value of a lottery requires that all parameters of a lottery are sequentially inspected before moving the attention to the other lottery. By contrast, anticipating regret requires more comparisons between lotteries, especially between the lowest monetary amount of one lottery and the highest of the other lottery. Thus, given that choices were mainly driven by *dEV*, we hypothesized that subjects would inspect amounts and probabilities within each lottery in every trial (intra‐lottery saccades). However, as choices were significantly modulated by anticipated regret as well, they displayed a secondary stereotypic visual pattern related to the evaluation of anticipated regret, which assumes more saccades between lotteries.

**Figure 4:**
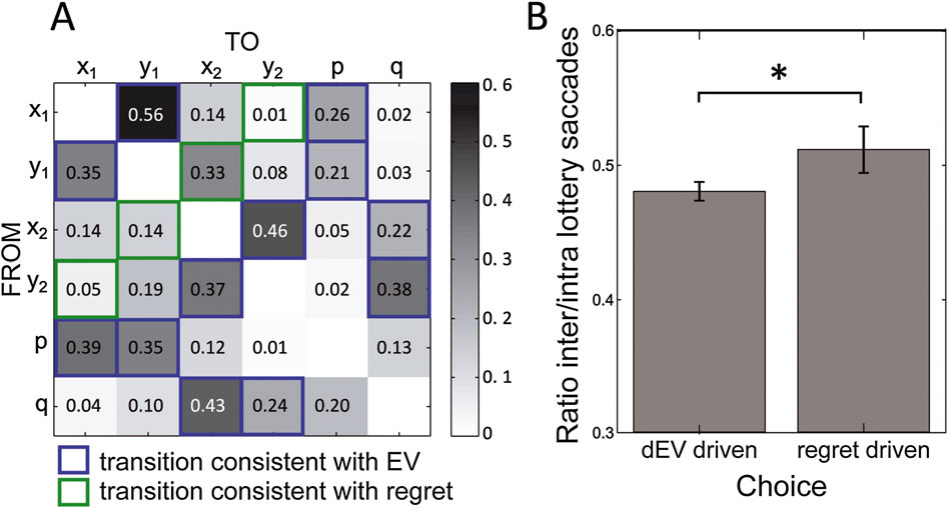
(A) Transition matrix. Each line corresponds to the starting AOI and each column to destination AOI. Numbers in the cells correspond to the proportion of transitions from the starting AOI to the destination AOI. Transitions relevant for the assessment of *dEV* and anticipated regret are indicated in blue and green, respectively. (B) Ratio of between and within lotteries saccades in expected value and regret‐driven choices. When minimizing regret rather than maximizing expected value, participants made more inter‐lottery saccades. Error bars stand for standard error of the mean

The transition matrix in Figure 4A shows the probability of participants looking from a particular AOI to any other AOI. Cells were not independent within each row (*χ* ^2^(30) = 12 549.69, *p* < .0001), suggesting that participants displayed consistent visual patterns. Three patterns emerged from this matrix. First, there were many transitions within lotteries, from *x* to *y*, *y* to *x*, and *x* or *y* to the associated probability. Second, participants preferentially explored elements of the lotteries from left to right, first *x*_1_ and then *y*_1_, *x*_2_, and *y*_2_. Finally, we observed some saccades consistent with regret evaluation, with transitions from *y*_1_ to *x*_2_ and from *x*_2_ to *y*_1_. The transition matrices were very similar in the two conditions, partial and complete feedback.

In order to further investigate the visual pattern of information acquisition, we defined three eye‐tracking variables, the total number of explored AOI during the decision phase of a trial, and the ratio of inter‐lottery and intra‐lottery saccades. Including those variable in our model of choice did not yield to significant results, possibly because of the much larger effect of *dEV* compared with *r* and *d* on decisions. Thus, we adopted an alternative approach. We compared regret‐driven decisions, where participants chose the lottery associated with minimal regret and with the lowest expected value (20.8% of the decisions), with expected value driven trials, where participants chose the lottery associated with the highest expected value (79.2% of the decisions). We tested the hypothesis that participants will attend monetary amounts more than probabilities and make more inter‐lottery comparisons when making decisions driven by regret. The number of number of entries into any AOIs was greater for trials in which decision was driven by regret (*M* = 16.6, *SEM =* .5) than for trials in which decision was driven by expected values (*M* = 14.5, *SEM =* .2, *t* = −4.12, *p* < .001, *d* = 1.10). This suggests that a conflict between the two decision criterions (*dEV* vs. *r*) may be quantified by additional attentional exploration. Our model of choice predicts that regret‐driven decisions are associated with inter‐lottery comparisons of monetary amount whereas *dEV*‐driven decision involves saccades within lotteries. To control for the difference in number of fixations between the two types of decisions, we compared the ratio of inter‐/intra‐lottery saccades between *dEV* and regret‐driven choices. As predicted, regret‐driven decisions involved a higher ratio of inter‐/intra‐lottery saccades (*M* = .51, *SEM* = .017) than *dEV*‐driven decisions (*M* = .48, *SEM* = .007; one‐tailed *t* = −1.786, *p* < .05, *d* = .49; Figure 4B). This result supports the idea that decision makers anticipate regret by comparing the potential outcomes of the different options, as opposed to attributing a value to each lottery independently from the alternative options.

#### Relationship between attention (eye‐tracking data) and behavior (choice data)

Regret theory proposes that comparing the outcome of a decision with the outcome of alternatives has a learning value. This entails that participants will seek counterfactual information more after a loss than after a gain and that it will affect their choice behavior. Participants, indeed, looked more at the outcome of the non‐chosen lottery (ON) on average when their choice yielded a negative outcome (OC < 0, *M* = 252.11 milliseconds, *SEM =* 16.85) than when it yielded a positive outcome (OC > 0, *M* = 206.60 milliseconds, *SEM =* 18.62; *t*(19) = 2.95, *p* < .01, *d* = .56). This result was confirmed by the mixed linear regression reported in Table 2, which shows, on a trial by trial basis, that decreasing obtained outcome increases the fixation duration on the outcome of the non‐chosen lottery. In addition, we found a significant correlation between the total fixation duration on ON and individuals' *r* coefficients extracted from the choice regression in complete trials (*r* = .54, *p* < .05), suggesting that participants who made more counterfactual comparisons during the feedback period are those who minimized regret the most when choosing.

**Table 2:** Mixed linear regression: the fixation time on the outcome of the non‐chosen lottery (ON) is estimated as a function of the obtained outcome (OC) and non‐realized outcome of the chosen lottery (NRC)

Fixation duration on ON	Coefficient	Standard error	*z*	*p*	95% confidence interval	
OC	−1.3770	.4885	−2.82	.005	−2.3345	−.4195
NRC	−.5356	.4713	−1.14	.256	−1.4594	.3881
Constant	230.0886	15.3424	15.00	<.001	200.0181	260.1590
Wald *χ* ^2^ = 8.01; Prob > *χ* ^2^ = .0182; complete trials						

Participants looked more at the outcome of the non‐chosen lottery (ON) when their obtained outcome (OC) decreased. The effect of the non‐realized outcome of the chosen lottery (NRC) was not significant.

#### Effect of experienced regret on the visual attentional pattern in subsequent choices

We also observed a change in visual inspection pattern over time. When we split the sample of eye‐tracking data acquired during choices into early (first 20 trials in each feedback condition) and late (last 20 trials in each feedback condition) trials, we observed that the result previously reported in Figure 4B—that is, that minimizing regret rather than maximizing expected value is associated with a higher inter‐/intra‐lottery saccade ratio—only holds in late trials (*t*(19) = 2.03; *p* < .05, *d* = .57). The difference in saccades type between *EV*‐driven and regret‐driven trials was not significant for early trials (*t*(19) = .14, *p* = .44). This suggests that the information acquisition pattern changes during the course of the experiment. After participants experienced regret (in early trials), they started (in late trials) examining the choice options by making more saccades between lotteries in an attempt to minimize future regret.

## Discussion

Our study builds on recent efforts to use eye‐tracking methodology, in conjunction with choice behavior analyses, to better capture the complexity of decision processes (Franco‐Watkins & Johnson, 2011; Glöckner & Herbold, 2011; Knoepfle, Wang, & Camerer, 2009; Krajbich, Armel, & Rangel, 2010; Krajbich & Rangel, 2011). We investigated how different decision variables, such as expected values and anticipated regret and disappointment, are evaluated and drive choices between lotteries. In particular, our goal was to test whether visual attention patterns were consistent with the predictions of our decision model based on regret theory.

During the decision period, we observed a fixation bias toward the chosen lottery, which is consistent with previous eye‐tracking results with binary choices (Krajbich et al., 2010; Shimojo, Simion, Shimojo, & Scheier, 2003). Analyses of choice behavior and visual pattern of information acquisition showed that participants seemed to primarily compare expected values of lotteries to make their choices, replicating previous findings (Fiedler & Glockner, 2012). Indeed, the most common visual pattern was to first look at all information (monetary amounts and probabilities) of one lottery and then switch to the other one. Nevertheless, anticipated regret modulated decisions as well, especially for those lottery pairs with small differences in expected values. In trials for which participants did minimize regret in their decision, they made more inter‐lottery comparisons than when their decision was driven by expected values. This study thus provides evidence for a regret‐related attentional pattern in decision making.

The feedback phase revealed fixation patterns clearly consistent with experienced regret during outcome evaluation. In the partial feedback condition, in which no information regarding the outcome of the non‐chosen lottery is provided, participants mainly looked at the non‐realized outcome of the lottery they had selected. In the complete feedback condition, participants spent a substantial amount of time looking at the outcome of the non‐chosen lottery and disregarded the non‐realized outcome of their lottery. These observations confirm that two distinct counterfactual outcomes serve as comparison points depending on the type of feedback provided. The visual exploration patterns are in accordance with subjective evaluation measures. In the partial feedback condition, the affective evaluation of the obtained outcome was modulated by the value of the non‐realized outcome of the chosen lottery. In the complete feedback condition, the affective evaluation of the obtained outcome was modulated by the value of the outcome of the non‐chosen lottery. This modulation was stronger in the complete feedback condition than in the partial one—the amplification effect—which is the signature of regret in this task.

Previous studies (Camille et al., 2004; Coricelli et al., 2005) showing that the non‐realized outcome of the chosen lottery has no effect on subjective evaluation have received important criticisms. The first criticism is that statistically disentangling the effect of the two types of counterfactual outcomes (the non‐realized outcome of the chosen lottery and the outcome of the non‐chosen lottery) on emotional ratings may be difficult. In a regret situation for instance, if the participant loses 20, both missed outcomes will be higher than the obtained outcome. This makes it difficult to ascertain that only the outcome of the non‐chosen lottery, and not both counterfactual outcomes, modulates emotional ratings of the obtained outcome, in the complete feedback condition. Second, emotional and attentional processes can have different effects. Even though the non‐realized outcome of the chosen lottery does not have any impact on emotional ratings, it could still be attended by participants and have a learning value (i.e., influence subsequent choices). Our model proposes an adaptive function of the emotion of regret; nonetheless, changes in choices behavior could happen outside of these emotional mechanisms. Therefore, the best way to address these criticisms is to investigate attentional patterns. The present eye‐tracking results provide additional insights for the interpretation of the subjective evaluation results. We show here that in the complete feedback condition, participants do attend the outcome of the non‐chosen lottery, all the more if their obtained outcome is a net loss. By contrast, the non‐realized outcome of the chosen lottery is only attended in the partial condition and ignored in the complete feedback condition. Thus, eye‐tracking data provide evidence that the non‐realized outcome of the chosen lottery does not influence the way participants evaluate their outcome when the non‐selected lotteries are resolved. These results are important to show that we can ignore disappointment in regret because both the subjective ratings and eye‐tracking data converge to the same findings.

These results are consistent with the proposed adaptive function of regret (Foster & Vohra, 1999; Foster & Young, 2003; Hart, 2005; Hart & Mas‐Colell, 2000; Megiddo, 1980): information about the outcome of rejected options serves as feedback on aptness of choice and as a learning signal to make better choices in the future. Therefore, in the complete feedback condition, participants attend counterfactual outcomes of the non‐selected option more than those of the selected option, because the former provide more useful information from a learning perspective. Consistently with this hypothesis, the eye‐tracking data showed also that participants looked more for counterfactual information (i.e., outcome of the non‐chosen lottery) in trials when they obtained a negative outcome. Additionally, participants who made more counterfactual comparisons during the feedback period relied more on anticipated regret in their decisions, also confirming the adaptive role of regret. These findings have important theoretical implications. Indeed, they show a hierarchy of counterfactual outcomes, with the outcome of the unchosen alternative weighted the most, thus demonstrating the relevance of theories, such as regret theory (Bell, 1982; Loomes & Sugden, 1982), which focus mainly on the outcome of alternative choices as the counterfactual term, and theories of learning in games (Hart & Mas‐Colell, 2000) based on regret. Those theories integrate the counterfactual term in a rational model of decision making.

Several eye‐tracking studies showed that decision maker do not rely on the deliberate computation of expected values (Arieli et al., 2011; Glöckner & Herbold, 2011). Nonetheless, whether decision makers spontaneously make use of heuristics and show attentional patterns consistent with these heuristics is subject to debate (Orquin & Mueller Loose, 2013). Combining econometrics and eye‐tacking methods allowed us to better characterize the decision processes involved in choices among lotteries. Individuals based their partially decisions on expected values. However, when the available options were difficult to discriminate based on their expected values, participants relied more on anticipated regret, and those decisions involve a pattern of attention consistent with the predictions of our model of choice.

In many real‐life decisions, we have the opportunity to learn about the outcome of alternative choices, and our results suggest that this is salient information. Indeed, participants spent almost as much time looking at the counterfactual outcome as at their own outcome. Our results suggest that when the outcomes of non‐chosen options are observable, counterfactual comparisons are deeply integrated in the decision process. Regret signals are pervasive in decision making; they are not simply biasing decisions, but they are fundamental constructs of the decision‐making process. Therefore, reinforcement learning models would benefit from incorporating a regret component, both at the stage in which the value function is updated though prediction error signals and at the stage in which the probability on actions is computed (Coricelli & Rustichini, 2010).

Interestingly, our data show that the experience of regret affected the way in which participants acquired visual information in subsequent choices; in particular, they increased the amount of inter‐lottery saccades (transitions) in late trials. These findings provide evidence for a dynamic interplay between emotions and attention in decision making. Thus, a specific pattern of attention characterizes the experience of regret during unfavorable outcomes, and then in turn experienced regret affects the pattern of visual information acquisition in subsequent (regret avoidance) choices. Unfortunately, we failed to uncover a clear link between attentional patterns and future choices on a trial by trial basis. We believe that the stronger effect of expected values comparison compared with anticipated regret on choices made it difficult to characterize this link. Future studies should ensure to keep the difference in expected values between choice options very small to better identify regret mechanisms. Furthermore, more research will be needed to characterize the precise mechanisms of regret learning, that is, how the experience of regret shapes future choice behavior.

## Supporting information

Table S1. Pairs of lotteries used in the experiment

Supporting info item
