# Supplementary material for: Different Attentional Patterns for Regret and Disappointment: An Eye‐tracking Study
Source: J Behav Decis Mak. 2016 Feb 9;29(2-3):194–205. doi: 10.1002/bdm.1938 (PMC6084306; doi:10.1002/bdm.1938)
Supplement: Supplementary file 1 — Supporting info item [file BDM-29-194-s001.doc]

# Appendix

| Trial | Condition | p | x_1_ | y_1_ | outcome lottery 1 | q | x_2_ | y_2_ | outcome lottery 2 |  | Trial | Condition | p | x_1_ | y_1_ | outcome lottery 1 | q | x_2_ | y_2_ | outcome lottery 2 |
| --- | --- | --- | --- | --- | --- | --- | --- | --- | --- | --- | --- | --- | --- | --- | --- | --- | --- | --- | --- | --- |
| 1 | partial | 0.5 | 20 | -5 | 20 | 0.2 | 20 | 5 | 5 |  | 41 | complete | 0.5 | 5 | -20 | 5 | 0.2 | 20 | -20 | -20 |
| 2 | partial | 0.8 | 20 | -20 | -20 | 0.5 | 20 | 5 | 20 |  | 42 | complete | 0.5 | 20 | -5 | -5 | 0.8 | 20 | -20 | 20 |
| 3 | partial | 0.2 | 20 | 5 | 5 | 0.8 | 20 | -5 | 20 |  | 43 | complete | 0.2 | 5 | -5 | -5 | 0.5 | 5 | -20 | 5 |
| 4 | partial | 0.5 | -5 | -20 | -5 | 0.2 | 5 | -20 | -20 |  | 44 | complete | 0.5 | 20 | 5 | 20 | 0.8 | 20 | -5 | 20 |
| 5 | partial | 0.5 | 5 | -20 | 5 | 0.2 | 20 | -20 | -20 |  | 45 | complete | 0.2 | 20 | 5 | 5 | 0.8 | 20 | -5 | 20 |
| 6 | partial | 0.8 | -5 | -20 | -5 | 0.2 | 5 | -20 | -20 |  | 46 | complete | 0.2 | 5 | -20 | 5 | 0.5 | -5 | -20 | -20 |
| 7 | partial | 0.8 | 20 | -20 | 20 | 0.5 | 20 | -5 | -5 |  | 47 | complete | 0.5 | 20 | -5 | -5 | 0.2 | 20 | 5 | 5 |
| 8 | partial | 0.5 | -5 | -20 | -5 | 0.2 | 20 | -20 | -20 |  | 48 | complete | 0.5 | 20 | 5 | 20 | 0.8 | 20 | -20 | -20 |
| 9 | partial | 0.5 | 5 | -20 | -20 | 0.2 | 5 | -5 | -5 |  | 49 | complete | 0.2 | 20 | -20 | 20 | 0.5 | -5 | -20 | -20 |
| 10 | partial | 0.8 | 20 | -5 | 20 | 0.5 | 20 | 5 | 5 |  | 50 | complete | 0.2 | 5 | -20 | -20 | 0.8 | -5 | -20 | -5 |
| 11 | partial | 0.5 | -5 | -20 | -20 | 0.2 | 5 | -20 | 5 |  | 51 | complete | 0.5 | 20 | 5 | 20 | 0.8 | 20 | -20 | -20 |
| 12 | partial | 0.8 | -5 | -20 | -5 | 0.2 | 5 | -20 | -20 |  | 52 | complete | 0.2 | 20 | 5 | 5 | 0.8 | 20 | -5 | 20 |
| 13 | partial | 0.5 | 20 | -5 | -5 | 0.8 | 20 | -20 | 20 |  | 53 | complete | 0.5 | -5 | -20 | -5 | 0.2 | 20 | -20 | -20 |
| 14 | partial | 0.2 | 5 | -5 | -5 | 0.5 | 5 | -20 | -20 |  | 54 | complete | 0.2 | 20 | -20 | -20 | 0.5 | 5 | -20 | 5 |
| 15 | partial | 0.8 | 20 | -5 | 20 | 0.2 | 20 | 5 | 5 |  | 55 | complete | 0.8 | -5 | -20 | -5 | 0.2 | 5 | -20 | -20 |
| 16 | partial | 0.5 | -5 | -20 | -20 | 0.2 | 20 | -20 | 20 |  | 56 | complete | 0.2 | 20 | 5 | 5 | 0.5 | 20 | -5 | 20 |
| 17 | partial | 0.8 | 20 | -5 | -5 | 0.5 | 20 | 5 | 20 |  | 57 | complete | 0.8 | 20 | -20 | 20 | 0.5 | 20 | -5 | -5 |
| 18 | partial | 0.2 | 20 | -20 | -20 | 0.5 | 5 | -20 | 5 |  | 58 | complete | 0.8 | 20 | -5 | 20 | 0.5 | 20 | 5 | 5 |
| 19 | partial | 0.2 | 20 | 5 | 5 | 0.5 | 20 | -5 | 20 |  | 59 | complete | 0.2 | 5 | -5 | -5 | 0.5 | 5 | -20 | -20 |
| 20 | partial | 0.5 | 20 | 5 | 20 | 0.8 | 20 | -20 | -20 |  | 60 | complete | 0.5 | -5 | -20 | -20 | 0.2 | 5 | -20 | 5 |
| 21 | partial | 0.5 | 5 | -20 | -20 | 0.2 | 5 | -5 | -5 |  | 61 | complete | 0.8 | -5 | -20 | -5 | 0.2 | 5 | -20 | -20 |
| 22 | partial | 0.5 | 20 | 5 | 20 | 0.8 | 20 | -20 | -20 |  | 62 | complete | 0.5 | 20 | 5 | 20 | 0.8 | 20 | -5 | -5 |
| 23 | partial | 0.5 | 20 | -5 | -5 | 0.2 | 20 | 5 | 5 |  | 63 | complete | 0.8 | 20 | -5 | 20 | 0.2 | 20 | 5 | 5 |
| 24 | partial | 0.8 | 20 | -20 | 20 | 0.5 | 20 | -5 | -5 |  | 64 | complete | 0.2 | 20 | 5 | 5 | 0.5 | 20 | -5 | 20 |
| 25 | partial | 0.5 | 20 | 5 | 20 | 0.8 | 20 | -5 | 20 |  | 65 | complete | 0.5 | 20 | -5 | -5 | 0.8 | 20 | -20 | 20 |
| 26 | partial | 0.2 | 5 | -20 | -20 | 0.8 | -5 | -20 | -5 |  | 66 | complete | 0.8 | 20 | -20 | -20 | 0.5 | 20 | 5 | 20 |
| 27 | partial | 0.2 | 5 | -20 | -20 | 0.5 | -5 | -20 | -20 |  | 67 | complete | 0.5 | 5 | -20 | 5 | 0.2 | 20 | -20 | -20 |
| 28 | partial | 0.5 | 5 | -20 | 5 | 0.2 | 20 | -20 | -20 |  | 68 | complete | 0.2 | 20 | -20 | 20 | 0.5 | -5 | -20 | -20 |
| 29 | partial | 0.2 | 20 | 5 | 5 | 0.8 | 20 | -5 | 20 |  | 69 | complete | 0.5 | 5 | -20 | -20 | 0.2 | 5 | -5 | -5 |
| 30 | partial | 0.2 | 20 | -20 | 20 | 0.5 | -5 | -20 | -20 |  | 70 | complete | 0.2 | 5 | -20 | -20 | 0.5 | -5 | -20 | -20 |
| 31 | partial | 0.8 | 20 | -5 | 20 | 0.2 | 20 | 5 | 5 |  | 71 | complete | 0.5 | -5 | -20 | -20 | 0.2 | 20 | -20 | 20 |
| 32 | partial | 0.2 | 20 | -20 | 20 | 0.5 | -5 | -20 | -20 |  | 72 | complete | 0.5 | -5 | -20 | -5 | 0.2 | 5 | -20 | -20 |
| 33 | partial | 0.5 | 20 | -5 | -5 | 0.8 | 20 | -20 | 20 |  | 73 | complete | 0.8 | 20 | -20 | 20 | 0.5 | 20 | 5 | 5 |
| 34 | partial | 0.2 | 5 | -20 | -20 | 0.8 | -5 | -20 | -5 |  | 74 | complete | 0.8 | 20 | -5 | 20 | 0.2 | 20 | 5 | 5 |
| 35 | partial | 0.2 | 20 | -20 | -20 | 0.5 | 5 | -20 | 5 |  | 75 | complete | 0.2 | 20 | -20 | -20 | 0.5 | 5 | -20 | 5 |
| 36 | partial | 0.2 | 20 | 5 | 5 | 0.5 | 20 | -5 | 20 |  | 76 | complete | 0.8 | 20 | -5 | -5 | 0.5 | 20 | 5 | 20 |
| 37 | partial | 0.2 | 5 | -20 | 5 | 0.5 | -5 | -20 | -20 |  | 77 | complete | 0.5 | 5 | -20 | -20 | 0.2 | 5 | -5 | -5 |
| 38 | partial | 0.2 | 5 | -5 | -5 | 0.5 | 5 | -20 | 5 |  | 78 | complete | 0.2 | 5 | -20 | -20 | 0.8 | -5 | -20 | -5 |
| 39 | partial | 0.8 | 20 | -20 | 20 | 0.5 | 20 | 5 | 5 |  | 79 | complete | 0.5 | 20 | -5 | 20 | 0.2 | 20 | 5 | 5 |
| 40 | partial | 0.5 | 20 | 5 | 20 | 0.8 | 20 | -5 | -5 |  | 80 | complete | 0.8 | 20 | -20 | 20 | 0.5 | 20 | -5 | -5 |

**Table S1. Pairs of lotteries used in the experiment.** x_1_ and y_1_ are the two possible outcomes of lottery 1; p is the probability of x_1_. The probability of y_1_ is 1- p. x_2_ and y_2_ are the two possible outcomes of lottery 2; q is the probability of x_2_.

| Choice | Coefficient | Std. Err | z | P | 95% cond interval | |
| --- | --- | --- | --- | --- | --- | --- |
| Difference in expected values *(dEV)* | 0.1799 | 0.0217 | 8.28 | <0.001 | 0.1373 | 0.2225 |
| Anticipated regret (*r*) | 0.0152 | 0.0054 | 2.8 | 0.005 | 0.0046 | 0.0258 |
| Difference in risk (*dsd*) | -0.0369 | 0.0138 | -2.68 | 0.007 | -0.0640 | -0.0099 |
| Feedback condition | -0.0114 | 0.1074 | -0.11 | 0.916 | -0.2218 | 0.1991 |
| Interaction *dEV* x condition | 0.0256 | 0.0300 | 0.85 | 0.395 | -0.0333 | 0.0844 |
| Interaction *r* x condition | 0.0084 | 0.0076 | 1.11 | 0.268 | -0.0065 | 0.0234 |
| Interaction *dsd* x condition | 0.0192 | 0.0197 | 0.98 | 0.329 | -0.0194 | 0.0579 |
| Interaction *dEV* x r | -0.0019 | 0.0014 | -1.33 | 0.185 | -0.0047 | 0.0009 |
| Interaction *r* x *dsd* | -0.0006 | 0.0005 | -1.1 | 0.271 | -0.0015 | 0.0004 |
| Interaction *dEV* x *dsd* | 0.0031 | 0.0034 | 0.91 | 0.362 | -0.0035 | 0.0097 |
| Interaction *dEV* x *r* x *dsd* | 0.0002 | 0.0003 | 0.85 | 0.396 | -0.0003 | 0.0007 |
| Constant | -0.0095 | 0.0853 | -0.11 | 0.912 | -0.1766 | 0.1576 |
| Log likelihood = -998.53; Wald chi²(6)= 191.22; Prob > chi² = 0.0000 | | | | | | |

Table S2. Mixed logistic regression analysis modeling choices. The probability of choosing the left lottery over the right one is estimated as a function of the difference in expected values between the two lotteries, anticipated regret and risk. The regression includes the interactions of these three variables with one another, and with the condition (partial vs. complete feedback).
